# The Potential use of Honey as a Remedy for Allergic Diseases: A Mini Review

**DOI:** 10.3389/fphar.2020.599080

**Published:** 2021-01-26

**Authors:** Poi Yi Aw Yong, Fahmida Islam, Hanis Hazeera Harith, Daud Ahmad Israf, Ji Wei Tan, Chau Ling Tham

**Affiliations:** ^1^ School of Science, Monash University Malaysia, Subang Jaya, Malaysia; ^2^ Department of Biomedical Science, Faculty of Medicine and Health Sciences, Universiti Putra Malaysia, Selangor, Malaysia

**Keywords:** allergic rhinoconjunctivitis, atopic dermatitis, allergic rhinitis, allergic asthma, allergy, honey, allergic fungal rhinosinusitis, mini review

## Abstract

Honey has been conventionally consumed as food. However, its therapeutic properties have also gained much attention due to its application as a traditional medicine. Therapeutic properties of honey such as anti-microbial, anti-inflammatory, anti-cancer and wound healing have been widely reported. A number of interesting studies have reported the potential use of honey in the management of allergic diseases. Allergic diseases including anaphylaxis, asthma and atopic dermatitis (AD) are threatening around 20% of the world population. Although allergic reactions are somehow controllable with different drugs such as antihistamines, corticosteroids and mast cell stabilizers, modern dietary changes linked with allergic diseases have prompted studies to assess the preventive and therapeutic merits of dietary nutrients including honey. Many scientific evidences have shown that honey is able to relieve the pathological status and regulate the recruitment of inflammatory cells in cellular and animal models of allergic diseases. Clinically, a few studies demonstrated alleviation of allergic symptoms in patients after application or consumption of honey. Therefore, the objective of this mini review is to discuss the effectiveness of honey as a treatment or preventive approach for various allergic diseases. This mini review will provide insights into the potential use of honey in the management of allergic diseases in clinical settings.

## Introduction

Allergic diseases, one of the most commonly occurring diseases worldwide, are a group of hypersensitivity disorder mediated by immunological mechanisms which can cause tissue damage and life-threatening reactions (Jutel and Akdis, 2011; Kaplan et al., 2012). Their prevalence continues to increase at an alarming rate across gender, age and racial groups, thereby increasing the global health burden substantially over the last 20 years in developed and developing countries (Pawankar et al., 2011). According to World Allergy Organization, allergies now affect up to 30%–40% of the population worldwide with children and young adults bearing the greatest burden of these diseases (Pawankar et al., 2011). Some of the common examples of allergic diseases include allergic rhinitis, asthma, conjunctivitis, atopic eczema, and life-threatening anaphylaxis. Several factors are considered to be responsible for the dramatic rise in allergic cases which include increase in pollution, climate change, reduction in biodiversity, urbanization of societies, and change in lifestyle and dietary habits (Pawankar et al., 2011). The current treatment available for allergic diseases include antihistamines (carbinoxamine; hydroxyzine), corticosteroids (beclomethasone; ciclesonide), biologics (omalizumab; dupilumab), and allergen immunotherapy. Each besets by several side effects such as lipodystrophy, glucocorticoid-induced osteoporosis, purpura, and rosacea (Schacke et al., 2002; Coondoo et al., 2014). The drawbacks of existing treatments for allergies has driven an interest in complementary and alternative medicine (CAM) as an alternative treatment. An example of CAM that has been receiving attention in modern medicine is honey.

Honey is a natural food substance produced from nectar and plant sweet deposits that is collected, processed and stored by bees. It is high in both nutritional and therapeutic values, and has been traditionally used to treat burns, wounds, cough, asthma, and several other gastrointestinal and cardiovascular problems (Eteraf-Oskouei and Najafi, 2013). Honey can be classified according to its source of nectar, i.e., floral or non-floral honey. Its color, thickness, aroma, taste and composition vary greatly depending on its source, the bee species, weather, geographical location, harvesting season and its processing and storage conditions (Subramanian et al., 2007; Silvano et al., 2014). Honey is a complex substance predominantly comprised of sugar, mainly fructose and glucose, and small quantities of other sugars such as maltose and sucrose (Khan et al., 2007). The protein content in honey ranges from trace amounts of amino acids such as proline, alanine, glycine (Hermosin et al., 2003) to enzymes such as catalase, amylase, invertase (Jeffrey and Echazarreta, 1996), which varies depending on the bee species that produce the honey. It has a very low content of vitamins and minerals, constituting only 0.02% of its weight (Alqarni et al., 2014). The average pH of honey is 3.9 (ranging from 3.4 to 6.1); this acidic pH is greatly attributed to the 0.57% organic acids, mainly, gluconic acid and citric acid present in honey (Gündoğdu et al., 2019). In terms of phytochemical composition, honeys like Tualang, Manuka, and Gelam have been shown to contain a high number of flavonoids and polyphenols such as quercetin, kaempferol, chrysin, and apigenin. The quantitative analysis for some of the phytochemicals have also been reported by other studies shown in Table 1. These phytochemical compounds have been reported to be responsible for the medicinal properties of honey such as anti-inflammatory, anti-oxidant, anti-microbial, anti-allergic, anti-diabetic, anti-cancer, anti-parasitic activity, anti-ulcer, wound healing, and cardiovascular disease prevention (Cornara et al., 2017; Samarghandian et al., 2017). Among these reported beneficial effects, anti-inflammatory, anti-oxidant, anti-cancer, and anti-microbial activities from various honey are well documented (Cornara et al., 2017). For example, apigenin, chrysin, and quercetin isolated from honey have been proven in one reported study to inhibit the growth of various bacterial species (Das et al., 2015), whereas isolated kaempferol from honey contains anti-cancer and anti-inflammatory activity (Hämäläinen et al., 2007; Ghaffari et al., 2012). However, very limited research had been conducted to study the anti-allergic properties of honey. Thus, this mini review aims to summarize the existing findings on honey and its potential as an anti-allergic agent. This review critically analyses the findings from both preclinical and clinical studies, and discusses the limitations and future prospects of honey in the management of allergy.

**TABLE 1:** The common reported phenolic and flavonoid compounds found in Tualang, Gelam, and Manuka honey as well as each of their respective quantitative data. design and study outcome of reported.

Author	Type of honey	Common reported phenolic and flavonoid compounds (µg/100 g honey)			
Sarfarz and Nor Hayati (2013); Khalil et al. (2011)	Tualang honey	Catechin	12.9–35.6		
		Caffeic acid	2.5–3.2		
		Benzoic acid	0.2–1.0		
		Naringenin	0.6		
		Trans-cinnamic acid	0.01–0.5		
		Gallic acid	0.4–0.4		
		Kaempferol	0.02–0.2		
		Syringic acid	0.02–0.1		
		p-Coumaric acid	0.04		
		Luteolin	ND		
		Hyacinthin	ND		
Putri Shuhaili et al. (2016); Hussein et al. (2011)	Gelam honey	Quercetin	1,588.9–1,594.3		
		Chrysin	1,498.6–1,504.6		
		Hesperetin	1,475.2–1,477.8		
		Gallic acid	859.4–876.8		
		Ellagic acid	558.8–575.7		
		Chlorogenic acid	502.8–528.1		
		Caffeic acid	428.8–442.0		
		Ferulic acid	356.9–381.4		
		p-Coumaric acid	301.5–308.3		
Alvarez-Suarez et al. (2014); Sarfarz and Nor Hayati (2013); Yao et al. (2003)	Manuka honey	Quercetin	180–550	4-Methoxybenzoic acid	ND
		Isorhamnetin	320–470	Abscisic acid	ND
		Chrysin	370–400	Methyl syringate	ND
		Luteolin	130–430	Phenyllactic acid	ND
		Pinocembrin	150–230	2-Methoxybenzoic acid	ND
		Kaempferol	130–260	(4-Methoxyphenyl)-acetic acid	ND
		Protocatechuic acid	45.7–53.7	Pinostrobin chalcone	ND
		Syringic acid	37–43	Desoxyanisoin	ND
		Genistic acid	24.7–36.1	Methyl syringate	ND
		Gallic acid	24.2–34.6	3,5-Dimethoxybenzoic acid	ND
		Benzoic acid	6.5–29.9	3-Phenyllactic acid	ND
		Protocatechualdehyde	6.3–20.1	Salicylic acid	ND
		Caffeic acid	17.2–18.6	Apigenin	ND
		Chlorogenic	16.1–16.9	Tectochrysin	ND
		p-Coumaric acid	ND	8-Methoxykaempferol	ND
		Myricetin	ND	Isoferulic acid	ND
		Pinobanksin	ND	Galangin	ND

ND, no data.

## CLINICAL STUDIES ON THE EFFECTS OF HONEY IN VARIOUS ALLERGIC DISEASES

### Atopic Dermatitis

A study conducted by Alangari et al. (2017) investigated the effectiveness of manuka honey on Atopic Dermatitis (AD) lesions by comparing the pathological status of honey-treated site to non-treated site. Three Item Severity score (TIS) was used to determine the degree of erythema, edema/papulation, and excoriation. Fourteen AD candidates from United Kingdom with bilateral similarly affected areas were requested to apply a layer of honey on the lesion site at night and wash it off the next morning for seven consecutive days. The authors reported that the honey-treated lesion had a significant improvement in the mean TIS score after 7 days of honey treatment. Interestingly, a 1 year follow up revealed that three of the participants reported an overall improvement in their eczema conditions without using honey after the study period, indicating that the beneficial effect of honey may retain in the skin even after stopping its topical application.

Another study reported by Al-Waili (2003) was a patient-blind clinical study where the authors compared the effects of natural honey obtained from Al-Theed City, UAE premixed with olive oil (honey mixture) in two groups of AD patients. The first group of patients consisted of 10 AD patients who did not receive any form of drug treatment before and during the study period. On the other hand, the second group of patients consisted of 11 AD patients who received corticosteroid treatment before and during the study period. All patients in the first group were subjected to treatment of honey mixture only whereas all patients in the second group were subjected to treatment of both honey mixture and corticosteroid ointment in different v/v ratios: mixture A (1:1), mixture B (2:1) and mixture C (3:1). From this study, eight out of 10 AD patients in the first group showed a significant improvement in AD symptoms after 1-week topical application of honey mixture (Table 2). Meanwhile, the patients who were in the second group showed a successful reduction in their dependence on corticosteroid at a range of doses by at least 50%. Although this clinical study demonstrated that topical application of honey mixture was able to relieve the symptoms of AD as well as decreasing the dependency of AD patients on corticosteroid, whether the olive oil itself exerted any anti-allergic properties was not demonstrated nor discussed. This is relevant since more recent studies have reported anti-allergic effects of olive oil (Isoda et al., 2012; Wani et al., 2015). Thus, the outcome of the study by Al-Waili (2003) could significantly be improved by an additional control group where the patients receive olive oil alone. This would be able to address any possible anti-allergic activities exerted by olive oil in the honey mixture. Not only that, the absence of a control group with corticosteroid only as the treatment in the second group may make the data interpretation to be difficult. Moreover, the number of participants in each subgroup was small (three to four patients per subgroup) if we were to consider the different dilutions utilized in the second group and therefore, the results obtained may not be generalizable. Finally, the insolubility of honey in olive oil might affect the outcome of this study.

**TABLE 2:** Shows summary of the disease model, experimental design and study outcome of reported clinical studies on the anti-allergic potential of various types of honey.

Author	Type of allergic disease	Type of honey	Number of patients	Age and gender	Grouping and treatment method	Honey treatment frequency	Scoring scale on disease symptoms	Symptoms evaluated	Study outcome (improvement of disease symptoms)	Remarks
Alangari et al. (2017)	Atopic Dermatitis	Manuka honey	14	Gender: 8 Female; 6 Male Mean age: 23–43 years	All participants: topical application of honey over the skin lesions at night and cover it with gauze. Remove the covering and wash the site in the morning	Once/day for 1 week	0 to 9 points (Three Item Severity Score)	Erythema Edema/papulation Excoriation	Yes	NA
Al-Waili et al. (2003)	Atopic Dermatitis	Natural unprocessed honey	21	Gender: 4 Female; 17 Male Age range: 5–16 years	Group 1 (10 participants with no topical treatment during recruitment) Right side body skin lesions: topical application of Vaseline Left side body skin lesions: topical application of honey mixture Group 2 (11 participants with ongoing topical corticosteriods treatment during recruitment) Right side body skin lesions: topical application of Vaseline and betamethasone esters 0.1% Left side body skin lesions: topical application of honey mixture with corticosteroids ointment	Three times/day for 2 weeks	0 to 4 points	Erythema Scaling Lichenification Excoriation Indurations/papulation Oozing/crusting Pruritis	Yes	Most patients also successfully reduced their dose of corticosteroid in honey mixture
Asha'ari et al. (2013)	Allergic rhinitis	Tualang honey	40	Gender: 26 Female; 14 Male Age range: 20–50 years	Group 1 (20 participants): daily 10 mg of loratadine for 4 weeks Oral ingestion of honey-flavored corn syrup (placebo) for another 4 weeks Group 2 (20 participations): daily 10 mg of loratadine for 4 weeks Oral ingestion of Tualang honey at 1 g/kg body weight for another 4 weeks	Once/day for 28 days	0 to 4 points (Allergic Rhinitis and Its Impact on Asthma Classification)	Nasal blockage Rhinorrhea Hyposmia Nasal, eye and palatal itchiness Sneezing	Yes	NA
Thamboo et al. (2011)	Allergic Fungal Rhinosinusitis	Manuka honey	34	Gender: No data Age range: >19 years	All participants: Intranasal application of 2 ml 1:1 honey/saline mixture using a mucosal atomization device in selected nostril	Once/day for 30 days	0 to 9 points (Philpott-Javer Endoscopic Scoring System)	Mucosal oedema and polyps Presence of mucin	No	Only nine out of 34 patients showed improment
Rajan et al. (2002)	Allergic rhinoconjunctivitis	Local honey Clover honey	36	Gender: 24 Female; 12 Male Age range: 20–72 years	Group 1 (12 participants): Oral ingestion of locally collected honey Group 2 (12 participants): Oral ingestion of Clover honey Group 3 (12 participants): Oral ingestion of honey-flavored corn syrup (placebo)	Once/day for 30 weeks	0 to 3 points	Nasal symptoms: Runny nose Sneezing Itchy nose Post-nasal drip Stuffy/blocked nose Ocular symptoms: Sore eyes Swollen eyes Watery eyes Itchy eyes	No	NA

NA, Not applicable.

### Allergic Rhinitis

A randomized placebo-controlled trial was done by Asha’Ari et al. (2013) to study the inhibitory effects of Tualang honey against Allergic Rhinitis (AR). Forty patients were first treated with 10 mg of second-generation antihistamine (loratidine) once daily for the first 4 weeks, followed by oral honey treatment (1 g per kg body weight per day) or placebo for another 2 months. At week 0, four and eight of the study, the patients were assessed according to the Allergic Rhinitis and Its Impact on Asthma (ARIA) classification focusing on seven symptoms (Table 2). The authors concluded that ingestion of honey significantly improves the mean of total symptoms score between honey treated group and placebo group, suggesting a progressive amelioration of AR symptoms following consumption of Tualang honey. In fact, the cardinal symptoms of AR were significantly improved in these patients even after discontinuation of antihistamines. This was not observed in the placebo group whose improvement in AR symptoms declined after week 4, possibly due to the cessation of the antihistamine treatment. Thus, this study demonstrates that the ingestion of honey along with usual standard medication is beneficial in relieving the AR symptoms without any reported adverse effect.

### Allergic Rhinoconjunctivitis

A clinical study by Rajan et al. (2002) demonstrated the effect of honey consumption on rhinoconjunctivitis by comparing the effects of locally collected honey from Bristol, England and clover honey (from Lancaster, England). Thirty-six Allergic Rhinoconjunctivitis (ARC) patients were randomly assigned into three groups; the first and second group of participants were instructed to consume a locally collected honey and clover honey respectively, while the third group was given placebo with honey-flavoured corn syrup. All the patients were requested to consume one tablespoonful of honey or corn syrup once per day for 30 weeks. Ten main symptoms were tracked and scored in this study which consisted of both nasal and ocular symptoms (Table 2). At the end of the study, no significant improvement in terms of symptoms score was found in the two honey treatment groups in comparison to the placebo group. This is thought to be due to inadequate dosage of honey to relieve the symptoms. The authors also highlighted that the dosage used caused 1/3 of the volunteers to withdraw from the study due to the unpleasant taste of the honey to consume on a daily basis.

### Allergic Fungal Rhinosinusitis

In a clinical trial performed by Thamboo et al. (2011), 34 Allergic Fungal Rhinosinusitis (AFRS) patients who had undergone bilateral functional endoscopic sinus surgery (FESS) and exhibited the main symptoms of AFRS were recruited. The patients were requested to spray 2 ml of manuka honey-saline solution (1:1 ratio) in their selected nostril once a day for 30 days, and those who were on prescribed medication were instructed to take their medication before spraying the honey solution. All patients acted as their own control as they continued with their current medical management in both nostrils, but only one nasal cavity was selected to undergo honey treatment. The evaluation was performed using Philpott-Javer Endoscopic Scoring System, which mainly grades the symptoms based on the presence of mucin, mucosal oedema, and polyps. The study demonstrated no significant improvement as only nine out of 34 patients receiving honey treatment showed positive response upon completion of the study.

### Clinical Prospect of Honey in the Treatment of Allergic Diseases

Although limited, the clinical evidence described above suggests that honey has great potential in the management of AD and AR. The general symptoms of AD such as erythema, excoriation and oedema can be improved significantly by topical application of honey, without major adverse effect. (Al-Waili, 2003; Alangari et al., 2017). However, the exact dosage of honey was not well-defined in these studies because the honey was either applied as a thin layer on the lesion site, or the honey mixture was prepared with other ingredients such as olive oil. Although the consumption of honey daily for 4 weeks was also reported to improve symptoms of AR. (Asha’Ari et al., 2013), it is still difficult to compare the effects of honey that used different treatment methods (oral ingestion, intranasal, and topical application) across different studies, regardless of the allergic diseases. Therefore, all these factors should be taken into consideration in any future studies as it may contribute to discrepancy when comparing the effectiveness of honey against various allergic diseases.

Although remarkable improvements were observed in certain allergic cases such as AD and AR, other studies reported no significant inhibitory effects of honey in some allergic diseases such as AFRS and ARC (Table 2), suggesting that honey might only be effective against certain types of allergic diseases. Even though honey had been reported to have no significant effect on AFRS, it is interesting to note that those AFRS patients who responded positively with honey treatment had been shown to have high level of specific IgE (Thamboo et al., 2011). Such observation can be explained by another study where honey has been shown to contain specific IgE lowering effect (Duddukuri et al., 1997), resulting in its ability to exert an inhibitory effect against AFRS through suppression of IgE levels in the patients. Nevertheless, further research will need to be conducted on these AFRS patients to identify the possible factors that contribute to their positive responses. Any future studies on the effects of honey on AFRS may consider to divide the patients into two groups (Group 1: with honey treatment; Group 2: without honey treatment) in comparison to the current experimental design of Thamboo et al. (2011), where the patients acted as their own control and only one nasal cavity was selected to undergo honey treatment. Based on the literature review, several studies provided limited information on the type of honey used. For instance, the clinical study on ARC and AD by Rajan et al. (2002) and a clinical study on AD by Al-Waili (2003) investigated the effect of a local honey but additional details such as the species of bee or the flora sources which the bees collect the nectar to produce the honey were not declared. This additional information may provide a better explanation for the outcomes of any related studies as there is evidence that these factors can influence the therapeutic effects of honey (Liu et al., 2013).

## Preclinical Studies on the Effects of Honey

### In vitro Model of Mast Cell Degranulation

A study demonstrated that manuka honey is able to inhibit allergic disease by modulating mast cell response (Alangari et al., 2017). In the study, the LAD-2 human mast cell line induced by calcium ionophore was used as an *in vitro* model of allergic reaction to measure the inhibition of histamine release, a key indicator of mast cell degranulation. They reported that pretreatment of honey (0.5, 1, and 2%) was able to inhibit the release of histamine in a concentration-dependent manner.

### Specific IgE Lowering Effect

A study conducted by Duddukuri et al. (1997) showed that intraperitoneal (i.p.) administration of 100 µl Rock bee honey (*Apis dorsata*) inhibited antibody responses in BALB/c, C57BL/6, and SWR/J mice induced by 10 µg OVA. Specifically, its anti-passive cutaneous anaphylaxis was indicated by the suppression of antigen-specific IgE levels (IgE titer <4). The fact that this effect was observed in different mice strains with different haplotypes highlights that the specific IgE lowering effect of Rock bee honey is less likely to be influenced by genetic variation. In addition, the authors also demonstrated that the Rock bee honey was able to inhibit the antigen-specific IgE levels in BALB/c mice induced by 100 µg of cowpea or finger millet, suggesting that honey treatment can even dampened the humoral antibody response following exposure to different types of allergen. Apart from Rock bee honey, the study also showed that Apiary and Dabur honey obtained from West Bengal, India were effective at downregulating the antigen-specific IgE production in OVA induced-BALB/c mice. This demonstrates the potential lowering effect of antigen-specific IgE in other Indian commercial honey. However, in contrast to rock bee honey, the authors did not specify any details on the Apiary and Dabur honeys used in their study, which are important to make a better justification on the positive outcome of the findings.

### Allergic Asthma

The beneficial effects of honey in allergic asthma have been shown in both mice and rabbit models where previous studies have supported that honey is a promising candidate to treat allergic asthma. The anti-asthmatic effect of raw Gelam honey (*Apis mellifera*) which originated from Malaysia has been reported previously (Shamshuddin and Mohd Zohdi, 2018). This study showed that oral administration of Gelam honey (40% and 80% (v/v)) exhibits a significant dose-dependent reduction in the airway epithelium thickening and infiltration of inflammatory cells (lymphocytes, neutrophils, and eosinophils) at peribronchiolar region and in the BALF of OVA-induced BALB/c mice. In addition, Gelam honey (10, 40, and 80% (v/v)) was also effective at attenuating the mast cells infiltration in the bronchial region and their beta-hexosaminidase production in the BALF, suggesting that this honey exerts anti-asthmatic effect by modulating the activities of mast cells, apart from other inflammatory cells. However, Gelam honey treatment only caused a marked reduction in airway mucin expression at 80% (v/v), an observation which the authors claimed is comparable to dexamethasone (3 mg/kg).

Another study investigated the effectiveness of aerosolized Tualang honey (25 and 50% (v/v)) as both rescue and preventative agents in OVA-induced rabbits (Kamaruzaman et al., 2014). Regardless of the dosage and treatment method (pretreatment or cotreatment), aerosolized Tualang honey was able to significantly inhibit goblet cell hyperplasia, mucus overproduction, and infiltration of inflammatory cells (eosinophils, mononuclear, neutrophils, and macrophage) in the peribronchial region and BALF in OVA-induced rabbits. These findings highlight the potential of Tualang honey in preventing allergic asthma as well as alleviating the symptoms. However, aerosolized Tualang honey only showed a significant decrease in the airway thickening of both epithelial and mucosal regions, but not in the submucosal region, regardless of the dosage and treatment methods. As submucosal thickening depends mostly on the innermost smooth muscle layer thickening (Kamaruzaman et al., 2014), it is speculated that aerosolized Tualang honey is unable to inhibit the thickening of smooth muscle layer within the airway.

On the other hand, a study conducted by El-Aidy et al. (2015) demonstrated the effect of Apiary honey, obtained from Dakahlia, Egypt, in a conalbumin-induced murine model of allergic asthma. The study reported that intraperitoneal administration of 650 mg/kg honey, along with intratracheal administration of conalbumin as the inducer, did not have any significant inhibitory effects in CD1 mice. In particular, there was no significant decrease in the number of infiltrated inflammatory cells (eosinophil, monocytes, neutrophils, and lymphocytes) within lung tissues when compared to the sensitized group.

## CONTROVERSIAL OUTCOMES FROM PRECLINICAL STUDIES ON THE USE OF HONEY AS AN ANTI-ALLERGIC AGENT

As described above, although limited, the preclinical studies mainly demonstrate that honey can significantly inhibit mast cell degranulation, anti-allergen IgE levels, as well as improve all the histopathological parameters of allergic asthma (Table 3). However, contradictory to all these positive results, the study conducted by El-Aidy et al. (2015) suggests that honey does not exert any anti-allergic effects. This discrepancy may be due to the variations between studies. For example, El-Aidy et al. (2015) used conalbumin as the inducer which was administered with the honey, whereas Shamshuddin and Mohd Zohdi (2018) induced the mice with ovalbumin. According to a previous study, conalbumin has a more faster response time and a stronger induction level compared to ovalbumin (Ho, 1979). These factors could influence the immune response and may partly explain the differential responses reported by these two studies. The findings reported by El-Aidy et al. (2015) may also be influenced by the route of administration. In the study, honey was administered intraperitoneally. A recent research has highlighted that the administration of honey by the aerosolized method allows it to readily deposit and be absorbed more easily in the airways (Abbas et al. 2019). Apart from that, similar with the clinical studies on ARC and AD, additional details on the honey used in the present study such as the bee species or the flora sources used by the bees to produce their honeys were not specified. This additional information may provide a better explanation for the negative outcomes of the study. More importantly, the bioactive compounds in honey have yet to be identified in previous studies. This may affect its anti-allergic activity as the chemical composition within the honey itself could give rise to different therapeutic responses (Rao et al., 2016).

**TABLE 3:** Pre‐clinical studies on the anti‐allergic potential of various types of honey.

Author	Type of allergic disease	Type of honey	Cell/Animal model	Number of animals	Age, gender, and weight	Treatment method	Control grouping	Honey treatment Grouping	Experimental outcome	Study conclusion (anti-allergic effects of honey)
Kamaruzaman et al. (2014)	Allergic asthma	Tualang honey	Rabbits (*Oryctolagus cuniculus*)	40 (5 in each group)	Mean age: ND Gender: 3 Female; 37 Male Mean weight: 2.40 ± 0.56 kg	Pre-treatment	Group 1: Normal control Group 2: i.p. injection with OVA (day 1 and 14) Group 3: i.p. injection with OVA (day 1 and 14), followed by aerosolised OVA (day 28–30) Group 4: i.p. injection with PBS (day 1 and 14), followed by aerosolised PBS (day 28–30)	Induction of allergic asthma was done with i.p. injection of OVA at day 1 and 14 Group 5: Aerosolised 25% (v/v) honey (day 23–27) Group 6: Aerosolised 50% (v/v) honey (day 23–27) Group 7: Aerosolised 25% (v/v) honey (day 23–27), followed by aerosolised OVA (day 28–30) Group 8: Aerosolised 50% (v/v) honey (day 23–27), followed by aerosolised OVA (day 28–30)	Inflammatory cell response (Wright-Giemsa stain): ↓ neutrophils, eosinophils, and macrophages infiltration in bronchoalveolar lavage fluid (BALF) Goblet cells (AB-PAS stain): ↓ cell hyperplasia and mucus accumulation in bronchioles	Yes
Shamshuddin and Mohd Zohdi (2018)	Allergic asthma	Gelam honey	Mice (BALB/c)	42 (6 in each group)	Mean age: 8–12 weeks old Gender: 42 Female Mean weight: ND	Pre-treatment	Group 1: Normal control Group 2: i.p. injection with OVA (days 0, 7, and 14), followed by intranasal instillations with OVA (day 14, 25, 26, and 27) Group 3: i.p. injection with OVA (days 0, 7, and 14), followed by intranasal instillations with OVA (day 14, 25, 26, and 27) and oral feeding of PBS Group 4: i.p. injection with OVA (days 0, 7, and 14), followed by intranasal instillations with OVA (day 14, 25, 26, and 27) and oral feeding of 3 mg/kg dexamethasone	Induction of allergic asthma was done with with i.p. injection of OVA at day 0, 7, and 14 Group 5–7: Intranasal instillations with OVA (day 14, 25, 26, and 27) and oral feeding with 10, 40 or 80% (v/v) of honey (day 14, 25, 26, and 27)	Histopathological analysis (hematoxylin and eosin (H&E) stain): ↓ eosinophil, neutrophils and lymphocytes infiltration in lung tissue Mucin expression (PAS stain): ↓ mucin expression in airway epithelium Mast cell count (toluidine stain): ↓ number of mast cells per bronchiole Total inflammatry cell count (wright-giemsa stain): ↓ number of inflammatory cells in BALF Beta hexosaminidase release assay: ↓ beta hexosaminidase release in BALF	Yes
El-Aidy et al. (2015)	Allergic asthma	Apiary honey	Mice (Albino CD1)	36 (6 in each group)	Mean age: 6 weeks old Gender: 42 Male Mean weight: 18–20 g	Co-treatment	Group 1: Normal control Group 2: i.p. injection with conalbumin (days 0 and 7), followed by intratracheal administration with conalbumin (day 14, 20, and 30) Group 3: i.p. injection with conalbumin (days 0 and 7), followed by intratracheal administration with conalbumin (day 14, 20, and 30) and i.p. injection with 0.5 mg/kg dexamethasone (day 15–33)	Group 4: i.p. injection with conalbumin (days 0 and 7), followed by intratracheal administration with conalbumin (day 14, 20, and 30) and i.p. injection with 650 mg/kg honey (day 15–33)	Histopathological analysis (H&E stain): No significant ↓ in eosinophil, monocytes, neutrophils, and lymphocytes infiltration in lung tissues	No
Duddukuri et al. (1997)	Passive cutaneous anaphylaxis	Rock bee honey	Mice (BALB/c, C57BL/6, and SWR/J)	ND	Mean age: 8 weeks old Gender: Female Mean weight: ND	Co-treatment	Group 1: Normal control Group 2–4: i.p. injection with OVA/cowpea/finger millet (days 0, 21, and 35)	Group 5–7: i.p. injection with OVA/cowpea/finger millet mixed with 100 µl rock bee honey (days 0, 21, and 35)	↓ IgE antibody level in the peripheral blood of all 3 species of mice	Yes
Alangari et al. (2017)	Mast cell degranulation	Manuka honey	LAD-2 (human mast cell line)	NA	NA	Pre-treatment	Group 1: Normal control cells Group 2 and 3: Cells induced with calcium ionophoreA23187	Group 3–5: Cells pre-treated with 0.5, 1 or 2% honey and later induced with calcium ionophore-A23187	Histamine EIA kit: dose-dependent ↓ in histamine release	Yes

ND, no data; NA, Not applicable.

**FIGURE 1:**
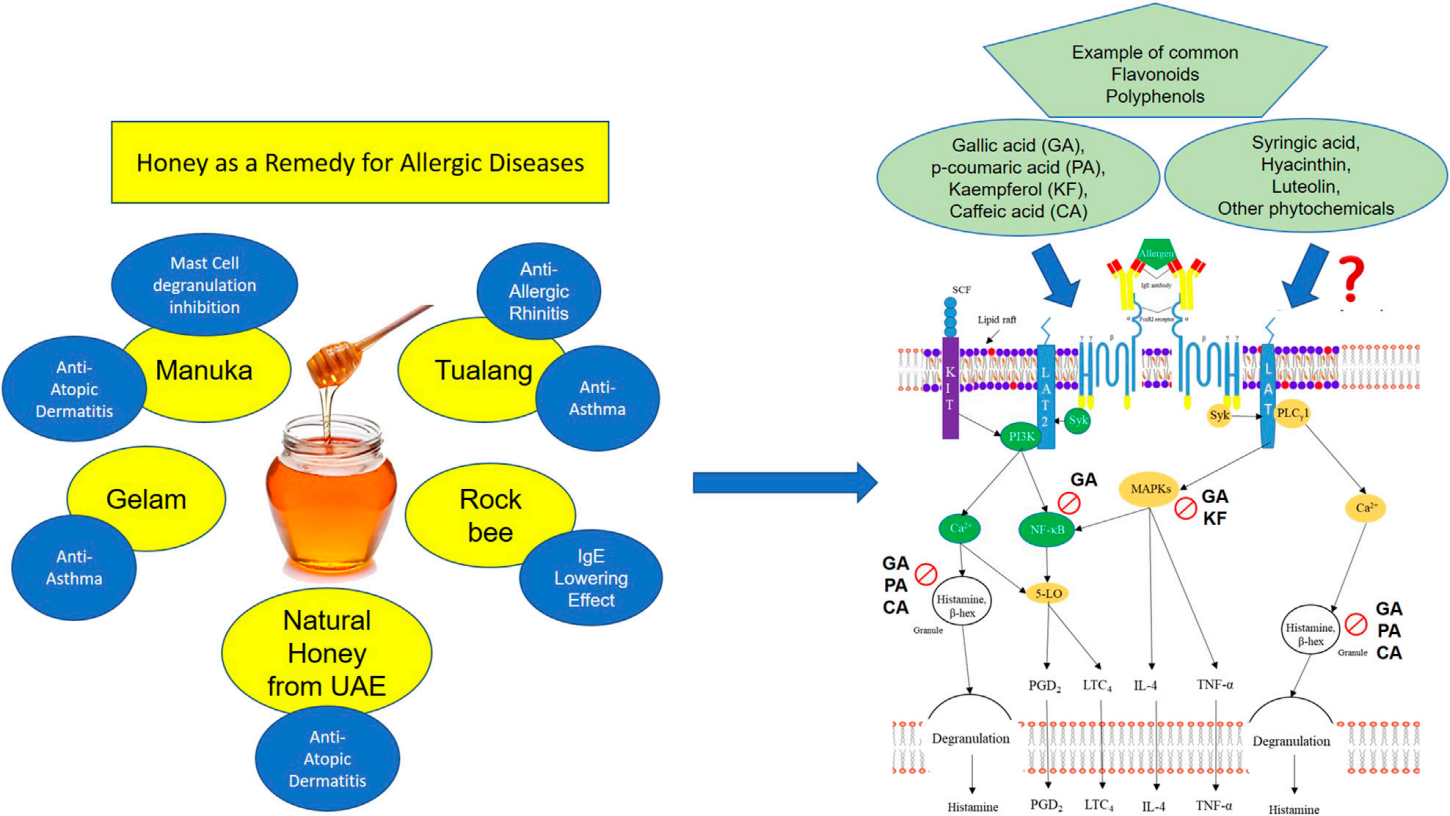
illustrates the association of phytochemical compounds present in honey (such as gallic acid, p-coumaric acid, kaempferol, caffeic acid, and etc.) which exhibit anti-allergic properties and their mechanism of action. Many of these commonly reported phytochemical compounds have been reported to exert their anti-allergic effects by inhibiting various mast cell degranulation signaling pathways. For example, gallic acid (GA) has been reported to inhibit NF-kB, MAPKs, and the release of granules containing histamine and β-hexosaminidase, which eventually leads to attenuation of allergic symptoms

Honey is known to contain various types of sugars and macronutrient; however, research mainly focuses on the biochemical compounds such as polyphenols which include flavonoids and phenolic acids which largely determine the bioactivity of honey. (Alvarez-Suarez et al., 2014; Putri Shuhaili et al., 2016; Ranneh et al., 2018). Although the general sugar composition of honey might be similar, the polyphenolic content may vary depending on various factors such as floral sources and local climate. Therefore, the identification of the bioactive compounds in honey as well as the inhibitory signaling pathways involved is necessary to provide a more complete insight on its mechanism of action against allergy. For example, clinical and preclinical evidence suggests that Manuka and Tualang honey may be good candidates in treating allergic diseases. Chemical composition analyses have shown that both Manuka and Tualang honey contain a vast number of phytochemicals which have been associated with their bioactivity including antioxidant, wound healing and anti-cancer properties (Alvarez-Suarez et al., 2014). Interestingly, both Manuka and Tualang honey share similar chemical composition including gallic acid, p-coumaric acid, kaempferol, syringic acid, and caffeic acid (Sarfarz and Nor Hayati, 2013; Alvarez-Suarez et al., 2014; Ranneh et al., 2018) and these bioactive compounds have been reported to demonstrate anti-allergic effects in other studies (Figure 1). For instance, Kim et al. (2006) reported that gallic acid inhibits the release of histamine, intracellular calcium and pro-inflammatory cytokines (TNF-α and IL-6) in rat peritoneal mast cells (RPMC) possibly via the regulation of nuclear factor-κB (NF-κB) and mitogen-activated protein kinase (MAPKs) activity. Besides, Lee et al. (2010) also reported that kaempferol exhibits anti-allergic properties through the suppression of β-hexosaminidase and cytokines (TNF-α and IL-4) release, as well as IL-4-induced activation of p38 MAPK in an *in vitro* model using RBL-2H3 cells (Lee et al., 2010). Another study conducted by Zhu et al. (2015) showed that p-coumaric acid and caffeic acid were able to inhibit the release of β-hexosaminidase through IgE-mediated RBL-2H3 cell degranulation. Nonetheless, there are many other phytochemical compounds identified in honey that are yet to be scientifically proven for its anti-allergic properties such as hyacinthin and luteolin. Furthermore, the possible synergistic effect of these phytochemicals in the attenuation of an allergic reaction can also be explored.

## Conclusion

In summary, this mini review summarizes the evidence on the effectiveness of honey in various allergic diseases in order to demonstrate the potential of honey as CAM. Although there is limited evidence, some studies showed remarkable improvements against certain types of allergic illnesses and support that honey is an effective anti-allergic agent. However, several research gaps remain to be filled, especially the identification of bioactive phytochemical compounds that are responsible for the anti-allergic effects of specific honey. Finally, more clinical studies are required to confirm the activity of honey in the pathogenesis of various allergic diseases and its mechanism of action to further justify its role as a future potential anti-allergic agent.

## Author Contributions

PA and FI prepared the manuscript. JT and CT conceived the idea, reviewed the drafts and provided important information for the completion. HH and DI reviewed the draft and provided important information for the completion. All authors approved the final version of this manuscript for submission.

## Funding

This study was supported by Fundamental Research Grant Scheme (FRGS) 2018 from Ministry of Higher Education of Malaysia (FRGS/1/2018/SKK06/MUSM/03/1).

## Conflict of Interest

The authors declare that the research was conducted in the absence of any commercial or financial relationships that could be construed as a potential conflict of interest.
